# Emerging insights on the role of gasdermins in infection and inflammatory diseases

**DOI:** 10.1002/cti2.1186

**Published:** 2020-10-04

**Authors:** Lipeng Tang, Chuanjian Lu, Guangjuan Zheng, Boudewijn MT Burgering

**Affiliations:** ^1^ Department of Pharmacology of Traditional Chinese Medicine The Second Affiliated Hospital of Guangzhou University of Chinese Medicine Guangzhou China; ^2^ Department of Molecular Cancer Research Center Molecular Medicine University Medical Center Utrecht Utrecht The Netherlands; ^3^ Department of Dermatology The Second Affiliated Hospital of Guangzhou University of Chinese Medicine Guangzhou China; ^4^ Department of Pathology The Second Affiliated Hospital of Guangzhou University of Chinese Medicine Guangzhou China

**Keywords:** Gasdermins, inflammatory bowel disease, multiple sclerosis, NETosis, pyroptosis, rheumatoid arthritis

## Abstract

The gasdermins, family of pore‐forming proteins, are emerging key regulators of infection, autoinflammation and antitumor immunity. Multiple studies have recently characterised their crucial roles in driving pyroptosis, a lytic pro‐inflammatory type of cell death. Additionally, gasdermins also act as key effectors of NETosis, secondary necrosis and apoptosis. In this review, we will address current understanding of the mechanisms of gasdermin activation and further describe the protective and detrimental roles of gasdermins in host defence and autoinflammatory diseases. These data suggest that gasdermins play a prominent role in innate immunity and autoinflammatory disorders, thereby providing potential new therapeutic avenues for the treatment of infection and autoimmune disease.

## Graphical abstract

This review summarises the latest advances in several aspects of mode of activation of gasdermins, which finally drives pyroptosis, NETosis, secondary necrotic death and apoptosis. Here, we also highlight the current understanding of gasdermin involvement in infections and autoimmunity disorders, providing a promising translational approach to the treatment of infectious and autoinflammatory diseases.

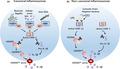

## Introduction

The gasdermins constitute a protein superfamily classified by the gasdermin domain and include six members [gasdermin A (GSDMA), gasdermin B (GSDMB), GSDMC, GSDMD, GSDME and Pejvakin (PJVK)]. The name gasdermin (gastro + dermato) is derived from the relative unique expression pattern of GSDMA in the upper gastrointestinal (GI) tract and the cutaneous epithelium.^1^ Besides the gasdermin domain, all gasdermin family members share approximately 45% overall sequence homology. At the structural level, gasdermins consist of two functional domains, the gasdermin‐N domain and gasdermin‐C domain. The N‐terminal domain (GSDM‐^NT^) of all gasdermin proteins, except PJVK, can oligomerise and therefore form membrane‐spanning pores in the plasma membrane leading to the release of inflammatory molecules, disruption of ionic gradients, osmotic cellular swelling and cytolysis, ultimately resulting in cell death. In healthy cells, however, the cytotoxic effects mediated through the N‐terminal domain can be suppressed by the autoinhibitory C‐terminal domain.^2^, ^3^, ^4^

In this review, we highlight recent advances in understanding the activation mechanism of the gasdermin. Moreover, we also focus on their roles in host immunity and autoinflammation diseases.

## The members of gasdermin family

### GSDMD

*GSDMD*, located at chromosome 8q24.2, is the best‐studied member of the gasdermin family. Initially, GSDMD was found to be expressed in the epithelial cells of the oesophagus and stomach.^5^ Recent studies revealed that GSDMD is also highly expressed in macrophages, neutrophils, monocytes, T cells and B cells,^6^, ^7^, ^8^, ^9^ where it acts as a key executor of inflammatory cell death.

GSDMD consists of a N‐terminal pore‐forming domain and a C‐terminal repressor domain, with a range of diverse cleavage sites for different caspases or granzymes within the linker region (Table 1). The crystal structure of GSDMD provides further understanding of the connection between these two domains. Liu and co‐workers demonstrated that two aromatic residues in β1‐β2 loop of N‐terminal domain (NTD; F50 and W51 in mouse GSDMD or F49 and W50 in human GSDMD) insert into a hydrophobic pocket consisting of L292, E295, Y376, A380, S470 and A474 in the C‐terminal domain. This hydrophobic pocket at the NTD‐CTD interface is conserved among members of the gasdermin family. Functional assays further indicated that this domain interface is critical for the autoinhibition of GSDMD.^4^

**Table 1:** Key cleaved residues of the gasdermin family

Key caspases/proteases	Gasdermin family	Key residues	Biofunctions	Reference
Caspase‐1	GSDMD	D275 in human being D276 in mouse	Cleavage by caspase‐1 leads to pyroptosis	6
Caspase‐4/5 (caspase‐11 in mouse)	GSDMD	D275 in human being D276 in mouse	Cleavage by caspase‐4,5 contributes to pyroptosis	10
Caspase‐8	GSDMD	D275 in human being D276 in mouse	Cleavage by caspase‐8 promotes pyroptosis	11, 12, 13, 14, 15
Caspase‐3	GSDMD	D87 in human being D88 in mouse	Cleavage by caspase‐3 counteracts pyroptosis	14, 15
Neutrophil elastase (NE)	GSDMD	C268 in human being V251 in mouse	Cleavage by NE results in NETosis	7, 16
Caspase‐4/5 (caspase‐11 in mouse)	GSDMD	D275 in human being D276 in mouse	Cleavage by caspase‐4,5 causes NETosis in neutrophils	58
Cathepsin G	GSDMD	L274 in mouse	Activation of GSDMD by cathepsin G drives inflammation in neutrophils	8
Granzyme A (GzmA)	GSDMB	Lys244	Cleavage by GzmA triggers pyroptosis	17
Granzyme B (GzmB)	GSDME	D270 in human being and mouse	Cleavage by GzmB induces pyroptosis	18
Caspase‐3	GSDME	D270 in human being and mouse	Cleavage by caspase‐3 initiates pyroptosis/secondary necrosis/apoptosis	18, 38 35, 62

Previous studies found that GSDMD can be cleaved by active caspase‐1 downstream of canonical inflammasome complexes, including NLRC4 inflammasome sensing of bacterial flagellin, NLRP3 inflammasome sensing of reactive oxygen species (ROS) and K^+^ and AIM2 inflammasome sensing of viral DNA.^6^ Additionally, GSDMD can also be cleaved by active caspase‐11 (caspase‐4/5 in humans) downstream of lipopolysaccharide (LPS)‐activated non‐canonical inflammasome complexes.^10^ Recently, the identification of GSDMD cleavage by caspase‐3/8,^11^, ^12^, ^13^, ^14^, ^15^ neutrophil‐specific elastase (ELANE),^7^, ^16^ cathepsin G^8^ and granzymes^17^, ^18^ has advanced our understanding of GSDMD activation and increased the number of possible activation mechanisms.

### GSDMA

Firstly, *GSDMA*, located at chromosome 17q21, was identified through its elevated expression in cells of GI tract and skin. Secondly, GSDMA expression appears silenced in primary gastric cancers and gastric cancer cell lines.^1^ These results suggest that GSDMA may play a critical role in suppressing carcinogenesis of gastric tissue. Later research indicated that transforming growth factor‐β (TGF‐β) indirectly upregulates GSDMA expression via induction of LIM domain only 1 (LMO1) in the gastric epithelial cell lines, which finally induces gastric epithelium cell apoptosis.^19^

By virtue of its expression in the epithelium of the skin, several studies have addressed the role of GSDMA in cutaneous biology. In mice, there exist three isoforms of GSDMA encoded by Gsdma1, Gsdma2 and Gsdma3. Mice with spontaneous or chemically induced mutations in Gsdma3 exhibited epidermal hyperplasia, hyperkeratosis and a hair‐loss phenotype in the skin.^20^, ^21^, ^22^ However, GSDMA3^−/−^ mice had no visible developmental skin abnormalities,^23^ suggesting that these mutations confer a gain of function. These hair‐loss‐related mutations were shown to abrogate the interaction between the C‐ and N‐terminal domains of GSDMA3, and in a later study, the N‐terminal domain of GSDMA3 was shown to induce pyroptosis.^6^ Together, the physiological function of GSDMA in the skin appears to be associated with the regulation of proliferation/ differentiation of epidermal stem cells and the hair follicle cycle.

### GSDMB

The human *GSDMB* gene, also located at chromosome 17q21, consists of 12 exons and has at least four alternatively spliced transcripts ranging in length from 1578 to 1646bp. However, there is no counterpart of the human GSDMB gene in the mouse genome.

GSDMB is highly expressed in certain tissues, particularly lung, oesophageal and gastric tissues.^5^, ^24^ The high expression of GSDMB in airway epithelial cells contributes to the pathology of asthma.^24^ Additionally, unlike the reduced expression of GSDMA, GSDMC and GSDMD in gastric and oesophageal cancers, GSDMB is overexpressed in gastric, uterine cervix and breast cancers, indicating that GSDMB may function as a potential oncogene.^25^, ^26^, ^27^ Given the high expression and potential tumorigenesis effects of GSDMB, targeting GSDMB might be a therapeutic strategy for gastric, uterine cervix and breast cancers. Moreno‐Bueno and co‐workers provided evidence that intracellular delivery of an antibody targeting GSDMB reduces aggressiveness of HER2‐positive breast cancer.^28^ Recently, Shao and colleagues indicated that cytotoxic lymphocytes, such as cytotoxic T cells (CTLs) and natural killer (NK) cells, can cause tumor cell death through GSDMB‐dependent pyroptosis, suggesting that GSDMB might be a good target to increase effectiveness of cancer immunotherapy.^17^

### GSDMC

*GSDMC*, located at chromosome 8q24.2, is also known as melanoma‐derived leucine zipper (MLZE) since its high expression in metastatic melanoma cells.^2^ However, it was first shown to function as an oncogene in colorectal cancer.^29^ GSDMC is expressed in the epithelium of the skin, and recently, it was shown that ultraviolet light (UV) can induce GSDMC expression in the skin via TRPV1/calcium/calcineurin/NFATc1 signalling.^30^ GSDMC in its turn upregulates MMP‐1, an important mediator of tissue damage by UV irradiation, through activating ERK and JNK pathways.^31^ However, in general, the functions of GSDMC remain poorly understood.

### GSDME

*GSDME*, located at chromosome 7p15, was originally identified as DFNA5 (deafness, autosomal dominant 5) since it is found mutated in familial ageing‐related hearing loss.^32^ Genetic mutations within intron 7 of human GSDME cause skipping of exon 8 and truncation of the autoinhibitory C‐terminus at residue 315, finally leading to hearing loss.^32^, ^33^ These observations suggest that the N‐terminal domain of GSDME, like the GSDMD N‐terminal domain, may possess spontaneous pore formation ability and therefore induce pyroptosis in cochlear cells.

GSDME expression is silenced in most cancer cells but expressed in many normal tissues. Identification of promoter hypermethylation and silencing of GSDME expression in colorectal cancer (CRC) suggests GSDME may function as a potential tumor suppressor for colon tumorigenesis.^34^ Consistent with its tumor suppressive role in CRC, GSDME deficiency accelerates melanoma growth.^35^ In addition, reduced GSDME levels are associated with worse 5‐year survival and increased metastases from breast cancers.^36^ Chemotherapeutics, such as cisplatin, lobaplatin and doxorubicin, were shown to trigger pyroptosis in cancer cell through caspase‐3‐dependent cleavage of GSDME.^37^, ^38^ Recently, GSDME has been found to exert a marked effect on the tumor immune microenvironment via pyroptosis.^18^, ^39^ Zhang and his group indicated that cytotoxic lymphocytes, including CD8^+^ T and NK cells, suppress tumor growth via GSDME. Mechanistically, granzyme B (GzmB) from killer cytotoxic lymphocytes induces GSDME‐dependent pyroptosis in tumor cells, by both directly cleaving GSDME and indirectly activating caspase‐3 to cleave GSDME.^18^ Additionally, Erkes and colleagues found that combined BRAF inhibitor and MEK inhibitor treatment causes GSDME‐mediated pyroptosis in *BRAF* ^V600E/K^ ‐mutant melanoma via active caspase‐3, ultimately resulting in HMGB‐1 release to activate antitumor T‐cell responses.^39^ Collectively, these studies define a new functional intersection between GSDME‐dependent pyroptosis and T‐cell/NK cell responses to tumor cells.

## Pejvakin

Firstly, *pejvakin* (*PJVK* or *DFNB59*), located at chromosome 2q31.1–q31.3, was identified in patients with auditory neuropathy.^40^, ^41^ Unlike other gasdermin members, PJVK has a truncated C‐terminal domain and lacks therefore the autoinhibitory function of this domain. Secondly, PJVK has not been shown to induce pore formation, yet has been shown to drive pexophagy, a selective form of autophagy that targets damaged peroxisomes.^42^ PJVK is accordingly broadly expressed in the membrane of peroxisomes in inner hair cells, can function as a ROS sensor and can recruit the autophagy machinery to selectively degrade peroxisomes (pexophagy) during exposure to loud noises. These results indicate that PJVK‐mediated pexophagy plays a critical role in reducing peroxisome proliferation, ultimately protecting auditory hair cells against noise‐induced oxidative stress damage.

## Gasdermin family members are involved in a diverse variety of mechanisms of programmed cell death

Pyroptosis, NETosis, apoptosis and secondary necrosis are four well‐studied modes of programmed cell death (PCD) that initially were considered to be independent of one another, but emerging evidence indicates that there is extensive crosstalk between these forms of PCD and that all four pathways can be activated by the same cell death effectors – the pore‐forming gasdermin proteins.

## Pyroptosis induced by GSDMD^NT^ via inflammatory caspases

Pyroptosis is a form of PCD that was originally described in 2000,^43^, ^44^ and is characterised by pore formations in the plasma membrane, swelling, rupture of the cell and release of cytosolic contents such as interleukin‐1 (IL‐1β), interleukin‐18 (IL‐18) and high mobility group box 1 (HMGB1). It is a form of necrotic cell death that has emerged as an important innate immune mechanism against intracellular pathogens, including *Escherichia coli*, *Salmonella typhimurium*, *Shigella flexneri* and *Burkholderia thailandensis*.^7^, ^10^, ^45^, ^46^

**Figure 1:**
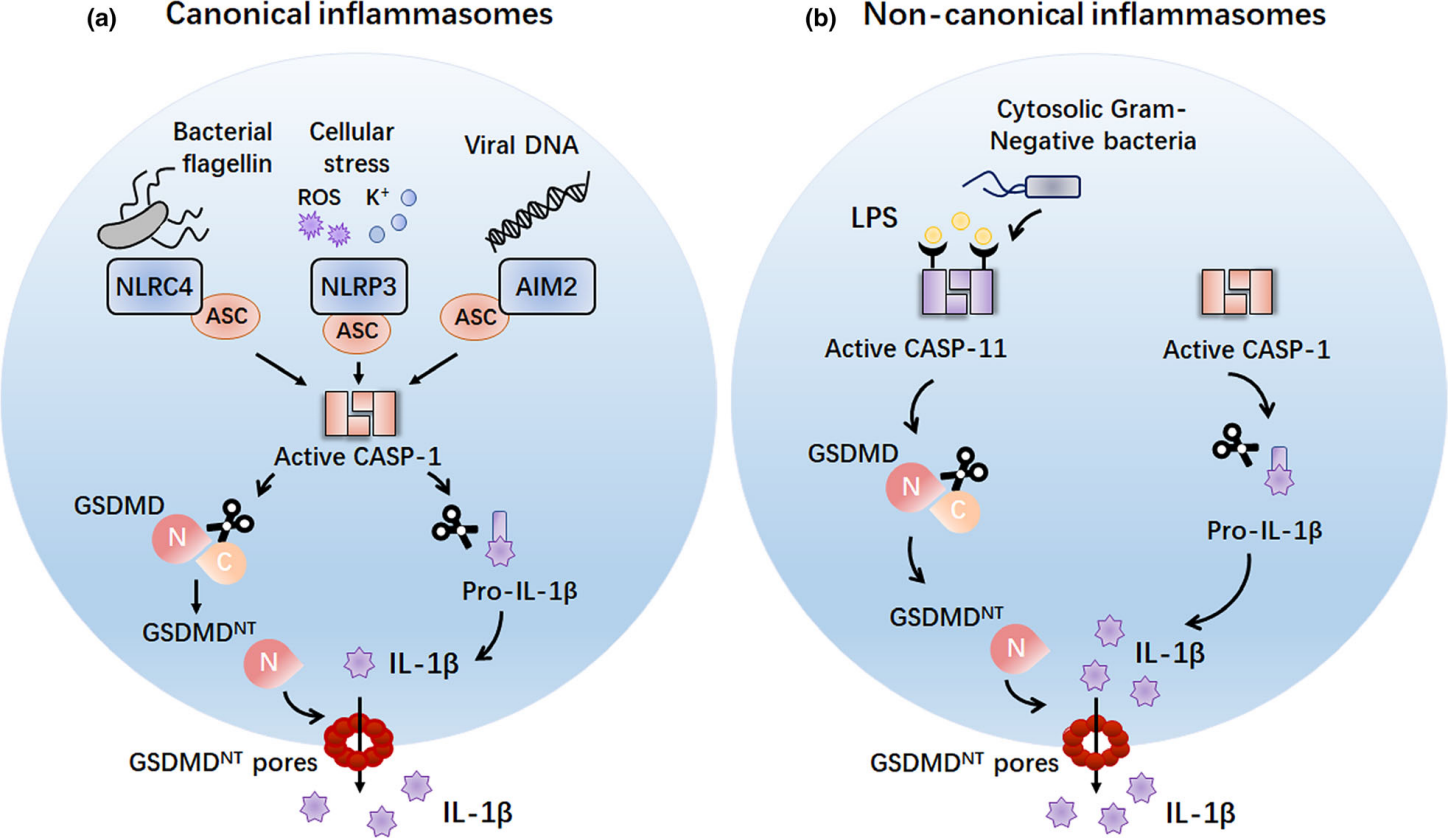
Inflammatory caspases induce GSDMD^NT^‐dependent pyroptosis. **(a)** The canonical inflammasome sensors, including NLRC4, NLRP3 and AIM2, detect diverse microbial signals and activate caspase‐1 through the ASC in macrophages. Active caspase‐1 can cleave GSDMD to liberate the pyroptotic GSDMD^NT^ domain to form pores in the plasma membrane, ultimately resulting in pyroptosis. In addition, active caspase‐1 also processes the pro‐inflammatory cytokine pro‐IL‐1β to generate mature IL‐1β, which is presumably released by cell lysis during pyroptosis. **(b)** The non‐canonical inflammasome detects cytosolic Gram‐negative bacteria or LPS molecules and activates caspase‐11 (or caspase‐4/5 in humans) in macrophages. Active caspase‐11 can cleave GSDMD to release the cytotoxic GSDMD^NT^ fragment to drive pyroptosis. ACS, apoptosis‐associated speck‐like protein containing a CARD; AIM2, absent in melanoma 2; CASP‐1, caspase‐1; CASP‐11, caspase‐11; LPS, lipopolysaccharide; NLRC4, NLR caspase activation and recruitment domain (CARD) domain‐containing 4; NLRP3, Nod‐like receptor (NLR) pyrin domain‐containing 3; ROS, reactive oxygen species.

Despite the important biological function of pyroptosis, the activation mechanism for pyroptosis remained unclear until recently. In 2015, three research groups independently and simultaneously, using different techniques (CRISPR/Cas‐9 nuclease screening, ENU‐forward mouse genetic screening and high‐sensitive quantitative mass spectrometry), demonstrated that GSDMD is a novel substrate of inflammatory caspase‐1 and caspase‐11 (caspase‐4/5 in humans), which cleave GSDMD between the N‐terminal domain (pore‐forming, pyroptotic‐inducing domain) and C‐terminal domain (autoinhibitory domain) downstream of canonical or non‐canonical inflammasome activation^6^, ^10^, ^47^ (Figure 1a and b). Recent structural insight into caspase‐1/4/11–GSDMD complex formation reveals that caspase‐1/4/11, which are autoprocessed after inflammasome activation, recognise and insert into a hydrophobic groove formed by Leu306, Leu310, Val367 and Leu370 of GSDMD‐C domain, ultimately cleaving GSDMD.^48^

Intriguingly, it has been shown that the N‐terminal domain of GSDMD (GSDMD^NT^), the cytotoxic fragment with approximate molecular weight of 30Kd (p30), which is released after caspase‐1/4/11 processing, selectively binds to phosphatidylinositol phosphates (PIPs), phosphatidylserine (PS) and phosphoinositide (PI; presence of which is restricted to the inner leaflet of the mammalian cell membrane) and cardiolipin (present in the inner and outer leaflets of bacterial membranes).^45^, ^49^, ^50^ Once GSDMD is cleaved, GSDMD^NT^ can bind to these lipids, oligomerise and form ring‐like pores (with an estimated inner diameter of about 10–16 nm) in the plasma membrane.^11^, ^45^, ^49^, ^50^, ^51^, ^52^ Using high‐resolution atomic force microscopy (AFM), Mulvihill *et al*. further revealed the mechanism of membrane pore formation by GSDMD^NT^. The time‐lapse AFM images indicated that GSDMD^NT^ can assemble arc‐, slit‐ and ring‐shaped transmembrane oligomers that insert into the PI‐, PIP‐ or PS‐containing membrane. Over time, these arc‐, slit‐ and ring‐shaped transmembrane oligomers can incorporate additional oligomers, thereby forming large and stable ring‐shaped oligomers. Gradual loss of membrane lipids in the inner side of these ring‐shaped structures ultimately generates complete transmembrane pores.^53^ Additionally, they observed no significant changes in the height of GSDMD^NT^ oligomers during GSDMD^NT^ pore formation, which demonstrates that the pore‐forming process of GSDMD^NT^ is initiated directly in lipid membranes without prepore‐to‐pore transitions.^53^, ^54^ Recent structural research further indicates that the lipid‐binding ability is dependent on the α1 helix and β1‐β2 loop of GSDMD^NT^.^4^ Additionally, three interfaces, including interface I (reverse‐parallel β3 and β8 strands), interface II (interface between α3 helix and β2 strand from one subunit and α2 helix and β11 strand from neighbouring subunit) and interface III (interface between α1 and α1′ helix from one subunit and α1 helix and β3 strand from adjacent subunit), are critical for the oligomerisation of the GSDMD^NT^ fragment.^4^ GSDMD‐mediated pore formation in the cellular plasma membrane finally results in the perturbation of electrochemical ion gradients and therefore causes cell swelling, rupture of plasma membrane and secretion of the cytosolic content, including proteins such as IL‐1β, IL‐18 and HMGB1. This feature of pore formation and release of cytosolic content is shared with GSDMA^NT^ and GSDME^NT^. These data identify gasdermin protein family members, GSDMA, GSDMD and GSDME, as the direct executors of pyroptotic cell death downstream of inflammatory caspase‐1 and caspase‐11.

## Pyroptosis triggered by GSDMD^NT^ and GSDME^NT^ via pro‐apoptotic caspases

**Figure 2:**
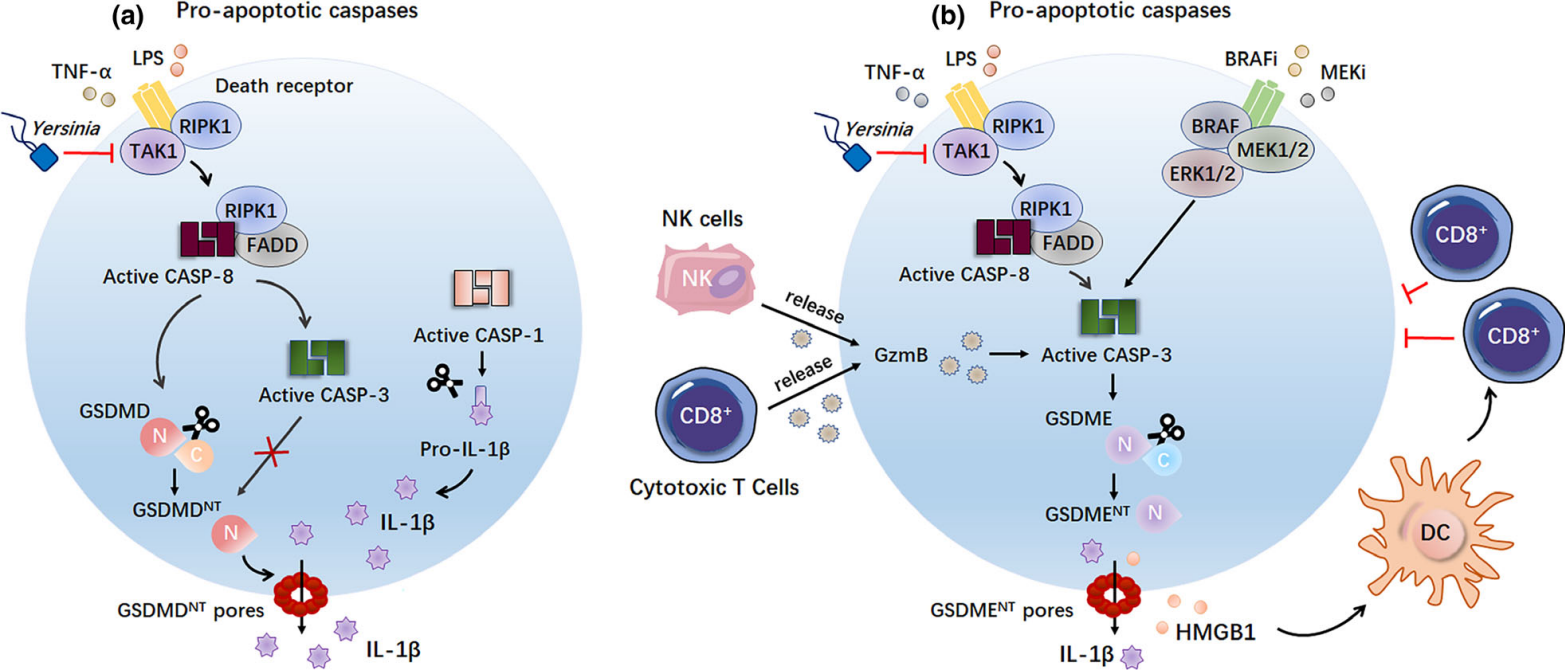
Pro‐apoptotic caspases trigger GSDMD^NT^‐ and GSDME^NT^‐mediated pyroptosis. **(a)** Caspase‐8, activated downstream of *Yersinia* infection or LPS/TNF‐α/TAK1 inhibitor co‐stimulation, cleaves GSDMD to generate the pyroptotic GSDMD^NT^ domain to trigger pyroptosis. Interestingly, caspase‐3, which is active downstream of caspase‐8 activation, inhibits GSDMDNT‐mediated pyroptosis by counteracting the cleavage of GSDMD. **(b)** Caspase‐3, which is active downstream of caspase‐8 activation upon *Yersinia* infection or LPS/TNF‐α/TAK1 inhibitor co‐stimulation, induces GSDME cleavage to trigger GSDME‐dependent pyroptosis in macrophages. In addition, both GzmB from killer cytotoxic lymphocytes and treatment with BRAF inhibitors and MEK inhibitors can induce caspase‐3 activation in cancer cells, finally leading to caspase‐3‐mediated GSDME‐dependent pyroptosis. BRAFi, BRAF kinase inhibitors; CASP‐1, caspase‐1; CASP‐3, caspase‐3; CASP‐8, caspase‐8; DC, dendritic cells; FADD, Fas‐associated protein with death domain; GzmB, granzyme B; HMGB1, high mobility group box 1; MEKi, MEK kinase inhibitors; RIPK1, receptor‐interacting protein kinase 1; TAK1, TGF‐β‐activated kinase 1.

Besides the inflammatory caspases, growing evidence suggests that pro‐apoptotic caspases, such as caspase‐8 and caspase‐3, also play a key role in regulating GSDMD activity in macrophages. Three independent studies revealed that caspase‐8 drives pyroptosis by inducing GSDMD cleavage.^12^, ^13^, ^14^ Sarhan *et al*., Orning *et al*. and Chen *et al*. observed caspase‐8, which is activated downstream of *Yersinia* infection or LPS/TNF‐α/TAK1 inhibitor co‐stimulation, cleaves GSDMD to generate active p30 fragments in macrophages, ultimately leading to pyroptosis (Figure 2a).

Interestingly, in addition to the active p30 fragment, it is also observed that GSDMD was cleaved into a p43 and p20 fragment upon *Yersinia* infection or LPS/TNF‐α/TAK1 inhibitor co‐stimulation. Chen *et al*.^12^ and Sarhan *et al*.^14^ suggested that caspase‐3, which is activated downstream of caspase‐8, counteracts the GSDMD‐dependent cell death by further processing the pyroptotic p30 fragment and full‐length GSDMD into the inactive p20 and p43 fragments, respectively (Figure 2a). Similarly, Taabazuing *et al*. provide evidence that caspase‐3 and caspase‐7 can also inactivate GSDMD by cleaving GSDMD at position D87 resulting into the p20 and p43 fragments.^15^ These results suggest that there is a balance between caspase‐8‐mediated GSDMD activation and caspase‐3‐mediated GSDMD inactivation. Future work should further explore how to regulate this balance upon infection or inflammation.

In contrast to GSDMD, GSDME can be cleaved via caspase‐3 in macrophages during *Yersinia* infection or LPS/TNF‐α/TAK1 inhibitor co‐stimulation. Additionally, as already mentioned, both GzmB from killer cytotoxic lymphocytes^18^ or combined BRAF inhibitor and MEK inhibitor treatment^39^ can trigger GSDME‐dependent pyroptosis in cancer cells downstream of caspase‐3 activation (Figure 2b). Interestingly, the cytokines released from pyroptotic cancer cells have a markedly promoting effect on the cytotoxic lymphocytes, giving a novel insight into the antitumor immunity.

## Pyroptosis caused by GSDMB^NT^ and GSDME^NT^ via granzyme

**Figure 3:**
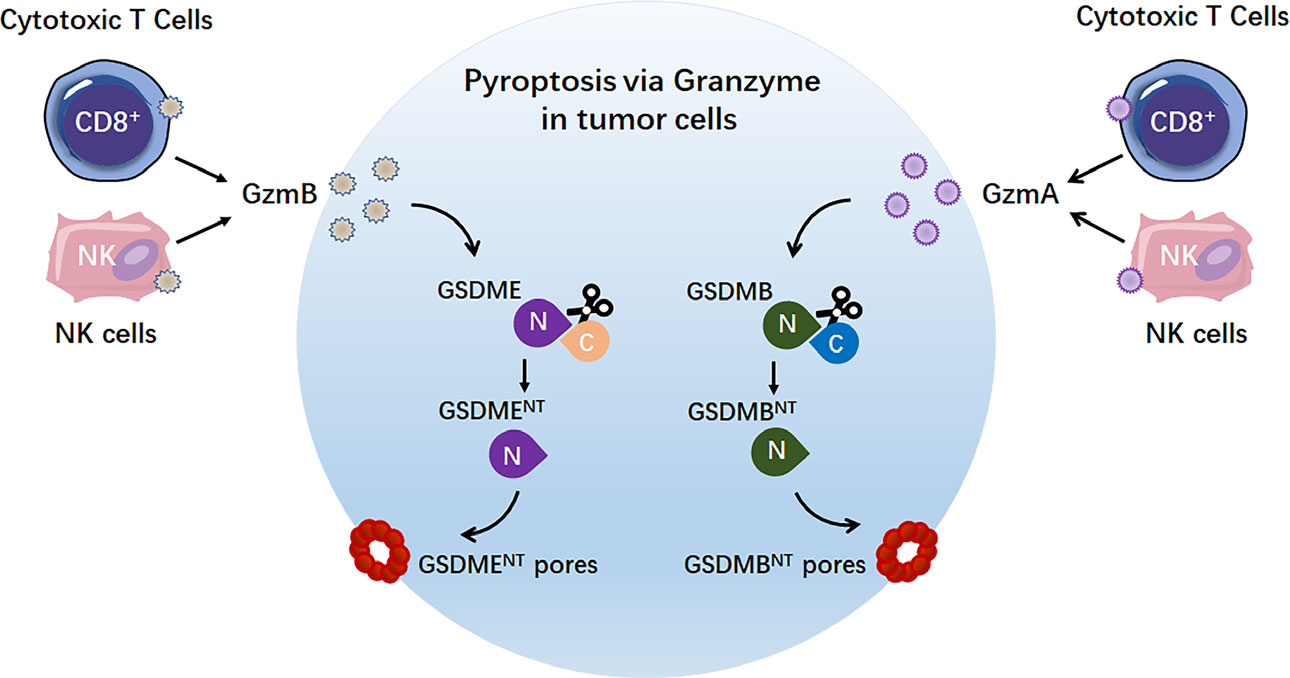
Granzymes initiate GSDMB^NT^‐ and GSDME^NT^‐induced pyroptosis. GzmA or GzmB, which is released from killer cytotoxic lymphocytes, induces GSDMB‐ or GSDME‐dependent pyroptosis in tumor cells, respectively. GzmA, granzyme A; GzmB, granzyme B; NK cells, natural killer cells.

In addition to caspases, gasdermins can be directly cleaved by granzyme. Indeed, Zhang *et al*.^18^ show that GzmB can directly cleave GSDME (Figure 3). This direct GzmB‐triggered pyroptosis provides a simple mechanism to induce pyroptosis without canonical or non‐canonical inflammasome activation.

Shao *et al*. extended this model by showing that granzyme A (GzmA) from NK cells and cytotoxic T lymphocytes, similar to GzmB, cleaves and activates GSDMB to induce cancer cell pyroptosis^17^ (Figure 3). These findings reinforce the view that pyroptosis can also be initiated in a less complex manner independent of caspases. Additionally, these data deepen our understanding of critical functions of gasdermin proteins in cytotoxic lymphocyte‐mediated tumor clearance.

## NETosis induced by GSDMD^NT^

Neutrophils, a specific type of white blood cell, function as an essential player of innate immunity directed against fungal and bacterial pathogens. NETosis, which is defined to be programmed neutrophil cell death, is an important antipathogen strategy employed by neutrophils. During NETosis, neutrophils can release chromatin, granular and cytoplasmic proteins and associated proteases to form an extracellular web‐like matrix, named neutrophil extracellular traps (NETs).^55^, ^56^ NETs can immobilise, catch and kill pathogens through their web‐like structures that consist of various antimicrobial proteins, including neutrophil elastase (NE), cathepsin G and histones.^57^ Given the key effects of NETosis on releasing antimicrobial NETs, NETosis is now recognised as a vital contributor to immune defence. However, the precise mechanisms of NETosis are not yet fully clear.

**Figure 4:**
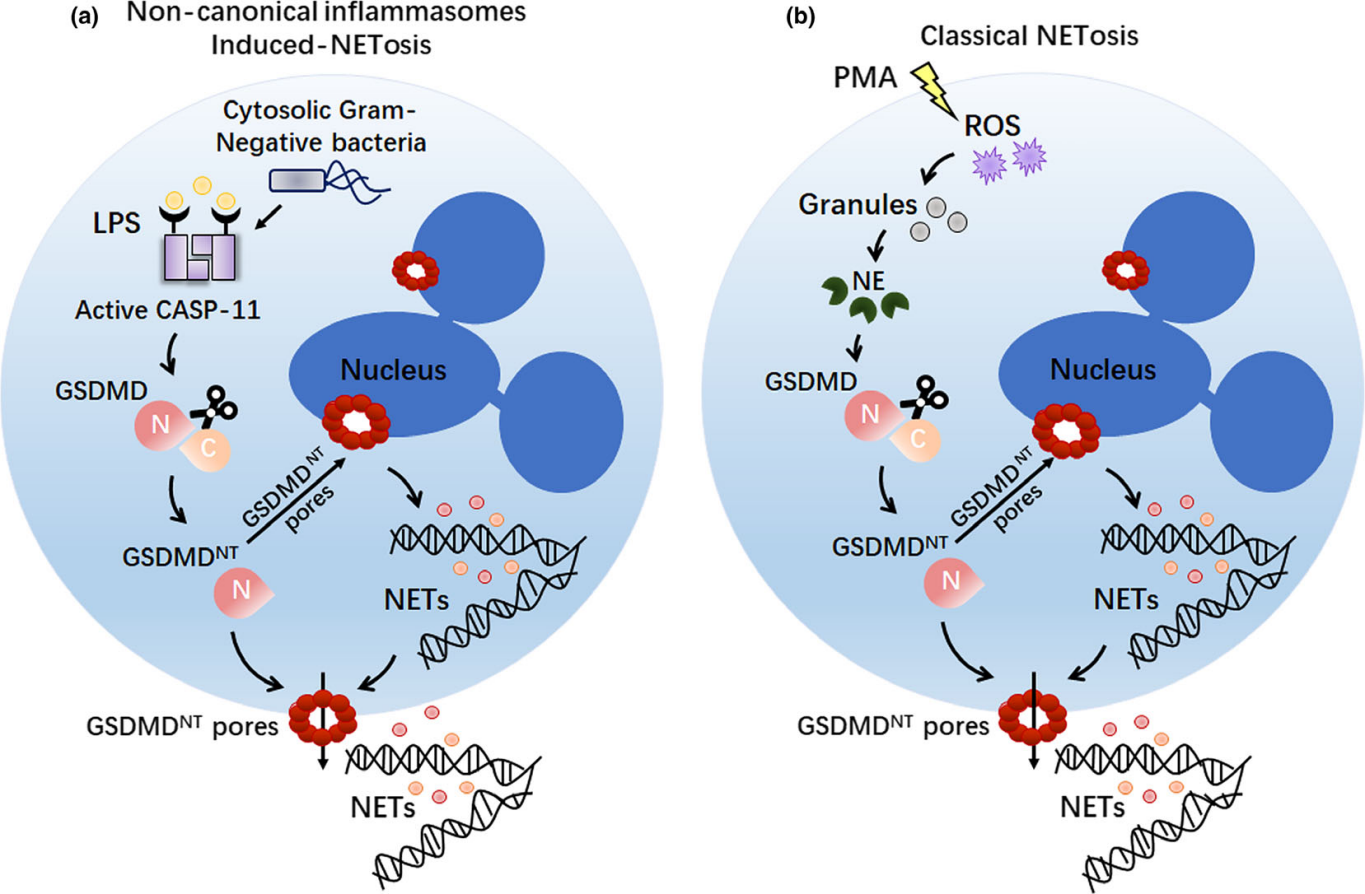
NETosis induced by pore‐forming GSDMD^NT^ fragment. **(a)** Upon cytoplasmic Gram‐negative bacterial infection or LPS stimulation, active caspase‐11 triggers robust GSDMD cleavage to generate GSDMD^NT^ domain. Then, the GSDMD^NT^ fragments form pores in nuclear and plasma membranes to NETs in neutrophils. **(b)** Upon treatment with classical NETosis activators (such as PMA), ROS induces NE release from the granules, leading to NE‐mediated GSDMD cleavage and activation. Cleaved GSDMD^NT^ fragments rupture nuclear and plasma membranes of neutrophil cells, resulting in NET extrusion. CASP‐11, caspase‐11; LPS, lipopolysaccharide; NE, neutrophil elastase; NETs, neutrophil extracellular traps; PMA, phorbol 12‐myristate 13‐acetate; ROS, reactive oxygen species.

Recently, Chen *et al*.^58^ found that cytosolic Gram‐negative pathogen or LPS triggers GSDMD‐dependent NETosis via a non‐canonical (caspase‐4/11) inflammasome signalling pathway (Figure 4a). However, neutrophils resist pyroptosis upon canonical inflammasome activation.^59^, ^60^ Interestingly, Sollberger and Hiroto Kambara found that GSDMD can be cleaved through a caspase‐independent way in the formation process of NET^7^, ^16^ (Figure 4b).

Taken together, these results provide a new avenue of understanding as to how GSDMD contributes to NET formation following diverse stimuli, including Gram‐negative pathogens, LPS, ROS or phorbol 12‐myristate 13‐acetate (PMA).

## GSDME^NT^ fragment switches apoptosis to secondary necrosis/pyroptosis

**Figure 5:**
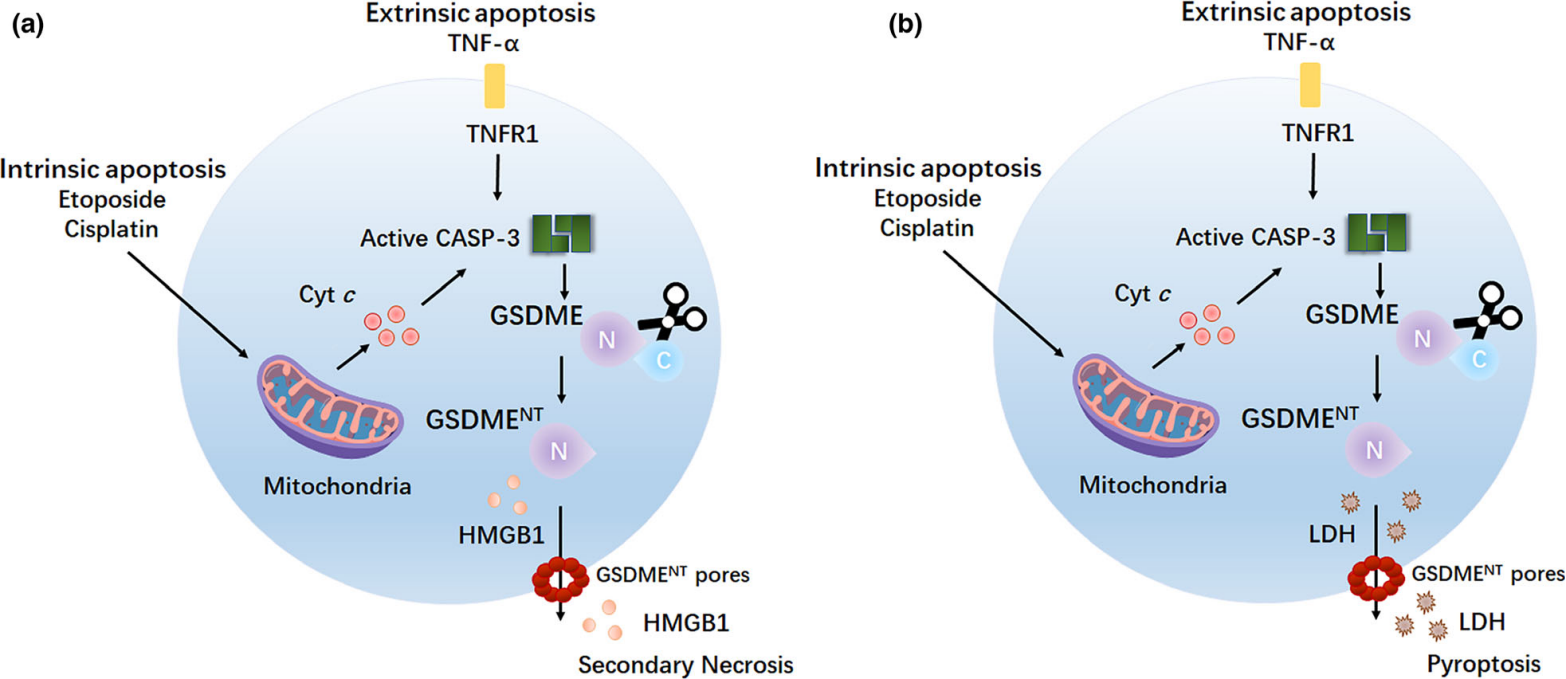
GSDME^NT^ switches apoptosis to secondary necrosis/pyroptosis. **(a)** Caspase‐3, which is activated by intrinsic or extrinsic apoptotic pathway, cleaves GSDME to generate the pyroptotic GSDME^NT^ fragment, which permeabilises the plasma membrane and releases pro‐inflammatory DAMP molecules such as HMGB1, finally leading to secondary necrosis in macrophages. **(b)** Caspase‐3, which is activated by intrinsic or extrinsic apoptotic pathway, mediates GSDME cleavage to generate the pyroptotic GSDME^NT^ fragment, which permeabilises the plasma membrane and releases LDH, finally leading to pyroptosis in cancer cells. CASP‐3, caspase‐3; Cyt *c*, cytochrome *c*; DAMP, danger‐associated molecular pattern; HMGB1, high mobility group box 1; LDH, lactate dehydrogenase.

Apoptosis is an immunologically silent form of PCD driven by caspase‐3/7/8/9 that typically results in rapidly engulfing dead cells by nearby phagocytes. However, in some instance, the scavenging of apoptotic cells is inhibited or insufficient in a timely manner, which causes these dying cells into a process called secondary necrosis. Secondary necrosis refers to a terminal phase at the end of the apoptotic programme characterised by plasma membrane permeabilisation, swelling, lysis and the release of intracellular pro‐inflammatory molecules including activated caspase‐3 and HMGB1.^61^ However, the molecular mechanism of secondary necrosis remains poorly understood. Emerging evidence indicates that secondary necrosis is orchestrated by the activity of apoptotic caspase‐3, which directly cleaves GSDME to produce a necrotic GSDME^NT^ fragment that targets and permeabilises the plasma membrane of macrophages^62^ (Figure 5a).

In addition to inducing secondary necrosis, GSDME can also switch caspase‐3‐mediated apoptosis to pyroptosis^18^, ^38^ (Figure 5b). As cancer cells express little GSDME, reversal of GSDME silencing can potentially sensitise cancer cells to chemotherapy drugs. Of caution here is that GSDME is highly expressed in various normal tissues, these GSDME‐mediated pyroptotic effects contribute to the extensive normal tissue damage and inflammation that occur in patients undergoing chemotherapy, and thus, lowering GSDME may be an alternative approach and a beneficial target for alleviating such adverse effects of chemotherapy drugs.

Taken together, these findings also extend our understanding of apoptosis. The expression level of GSDME may determine the form of cell death, including secondary necrosis or pyroptosis, upon ‘apoptotic stimulations’ in caspase‐3‐activated cells.

## Gasdermins augment the mitochondrial apoptotic pathway

**Figure 6:**
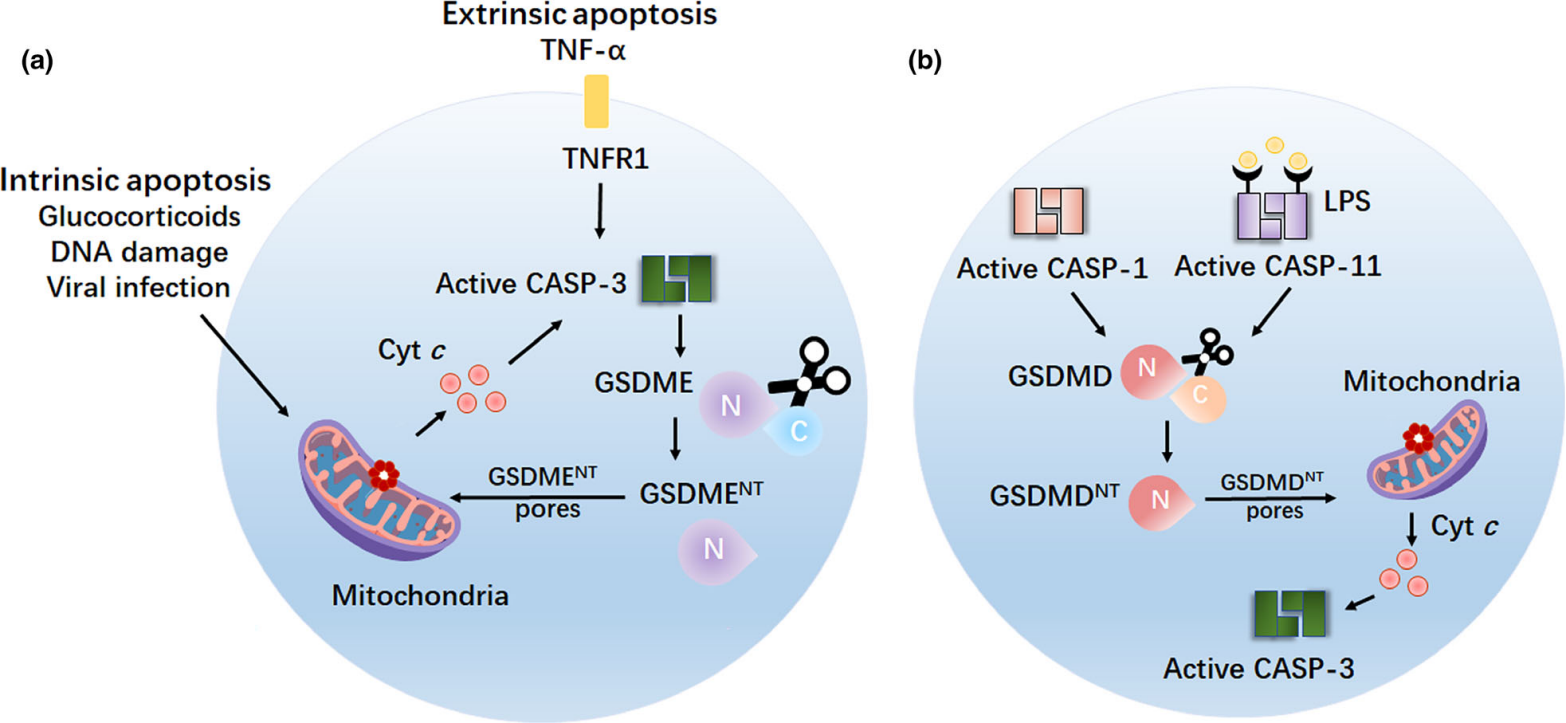
Gasdermins augment the mitochondrial apoptotic pathway. **(a)** Caspase‐3, which is activated by intrinsic or extrinsic apoptotic pathway, cleaves GSDME to generate the pyroptotic GSDME^NT^ fragment, which permeabilises the mitochondria membrane, releases cytochrome *c* (Cyt *c*) and activates the apoptosome in cancer cells and immortalised bone marrow‐derived macrophages (iBMDMs). Cyt *c* released through GSDME^NT^ mitochondrial pores, in turn, drives caspase‐3 activation and GSDME cleavage. **(b)** Canonical or non‐canonical inflammasome‐induced GSDMD^NT^ fragment can permeabilise the mitochondria and release Cyt *c*, finally enhancing the mitochondrial apoptotic pathway in cancer cells and iBMDMs. TNFR1, tumor necrosis factor receptor type 1; Cyt *c*, cytochrome *c*; CASP‐3, caspase‐3; CASP‐1, caspase‐1; CASP‐11, caspase‐11.

Interestingly, and complementary to the above, new evidence demonstrated that members of the gasdermin family also play additional roles in the apoptotic programme (Figure 6).

GSDME^NT^, which is cleaved by active caspase‐3, can also form pores in the mitochondrial membrane resulting in the release of pro‐apoptotic molecules, such as cytochrome *c* (Cyt *c*) and high temperature requirement protein A2 (HtrA2/Omi). This event creates a positive feedback loop that promotes caspase‐3 activation and further GSDME cleavage, ultimately augmenting the apoptotic programme^35^ (Figure 6a). The above mechanism might explain how the human deafness‐associated GSDME mutant induces cochlear cell death. Indeed, the absence of the gasdermin‐C domain in the GSDME hearing‐loss mutant leads to form pores in the mitochondria, release Cyt *c* and activate caspase‐3, finally resulting in cochlear hair cell apoptosis and sensorineural hearing loss. Furthermore, this function appears to be conserved within the superfamily, as other members such as GSDMA^NT^ and GSDMD^NT^ also permeabilise the mitochondria to release pro‐apoptotic factors^35^ (Figure 6b).

## Roles of Gasdermin family members in the host defence against microbial infection

In normal physiology, gasdermin family members play an important role in antimicrobial innate immune defence through pyroptosis, NETosis and direct killing of bacteria.

As mentioned above, active GSDMD, which is cleaved by caspase‐1 downstream of canonical inflammasome complexes, can restrict *Legionella pneumophila*, *Rotavirus* and *Francisella novicida* infections through pyroptosis in bone marrow‐derived macrophages and epithelial cells.^63^, ^64^, ^65^ Additionally, GSDMD‐dependent pyroptosis in bone marrow‐derived macrophages and monocytes, which is triggered through mouse caspase‐11 or human caspase‐4, is another critical host response against Gram‐negative bacteria, such as *E. coli*, *S. typhimurium*, *S. flexneri* and *B. thailandensis*.^10^, ^47^ Finally, these infective and pyroptotic macrophages functionally trap the bacteria, which results in pore‐induced intracellular trap‐mediated efferocytic clearance of bacteria by neighbouring macrophages and neutrophils *in vitro* and *in vivo*.^66^

In addition to pyroptosis, GSDMD can also exert host protective effects by promoting NETosis in neutrophils. GSDMD‐dependent neutrophil death, which is triggered by non‐canonical (caspase‐4/11) inflammasome downstream of cytosolic Gram‐negative bacteria, induces antimicrobial NET extrusion via forming pores in the nuclear and plasma membrane of neutrophils.^58^

Previous studies demonstrated that bacteria, such as *E. coli*, *Mycobacterium* and δ‐proteobacterium *Bdellovibrio bacteriovorus,* accumulate cardiolipin, PI and PS in the bacterial membrane.^67^, ^68^, ^69^ Given the selective lipid‐binding properties of the GSDMD^NT^ fragment, GSDMD^NT^ can bind to bacteria, which are enriched with cardiolipin, PI and PS in the membrane. Then, GSDMD^NT^ can directly and efficiently kill intracellular and extracellular bacteria by forming transmembrane pores and ultimately increasing permeabilities in the bacterial membrane.^3^, ^45^, ^46^, ^50^ Lipid‐specific binding may explain selective killing by GSDMD^NT^, yet different bacterial classes also differ in the composition and structure of their cell wall. The bacterial cell wall imposes a structural barrier for passage of gasdermin fragments. Whether and how, for example, GSDMD^NT^ passes the bacterial wall remains elusive, and thus, the apparent differential sensitivity of bacteria to gasdermin‐mediated killing is likely a combination of both cell wall composition and membrane lipid composition.

## Role of the gasdermin family in inflammatory diseases

GSDMD activation may in fact be a double‐edged sword in host responses. Excessive inflammatory cell deaths by gasdermin activations may play a detrimental role in host defence. In agreement, several studies demonstrated that gasdermin family members contribute to septic shock and autoinflammatory diseases, including multiple sclerosis (MS), systemic sclerosis (SSc), inflammatory bowel disease (IBD), rheumatoid arthritis (RA), familial Mediterranean fever (FMF) and type 1 diabetes (T1D; Table 2).

**Table 2:** The role of gasdermins in autoimmunity diseases

Autoimmunity diseases	Gasdermins	Study type	Biofunctions	Reference
Multiple sclerosis (MS)	GSDMD	Experimental evidence	GSDMD‐mediated pyroptosis inducing neuroinflammation via promoting and recruiting active T‐cell infiltration into the central nervous system (CNS)	78
Systemic sclerosis (SSc)	GSDMA	GWAS	The variant rs3894194, a missense mutation of GSDMA, is a novel susceptibility locus for SSc	79
Inflammatory bowel disease (IBD)	GSDMA GSDMB GSDMD	GWAS Experimental evidence	GSDMA and GSDMB were proved to be IBD susceptibility genes; GSDMD‐mediated pyroptosis contributes to IBD	80, 81, 82, 83
Rheumatoid arthritis (RA)	GSDMB GSDMD	GWAS Experimental evidence	GSDMB locus is associated with the risk of RA; GSDMD‐conferred monocyte pyroptosis is involved in RA	84, 85, 86
Familial Mediterranean fever (FMF)	GSDMD	Experimental evidence	GSDMD‐mediated pyroptosis is critical for autoinflammatory pathology in FMF	89
Type 1 diabetes (T1D)	GSDMB	GWAS	A risk allele of rs2290400 in GSDMB probably determines the risk of T1D predominantly in early childhood	90, 91, 92, 93

GWAS, genome‐wide association studies.

### Septic shock

Sepsis, which is defined as life‐threatening organ dysfunctions caused by a host's dysregulation following infection, is still a major challenge for healthcare systems. In general, pyroptosis protects multicellular host organisms against invasive pathogenic microbial infections; however, excessive pyroptosis might lead to an overwhelming inflammatory response, finally resulting in sepsis and septic shock. Emerging evidence suggests that GSDMD‐dependent pyroptosis induced by caspase‐1/11 inflammasome activation drives sepsis downstream of LPS exposure/TMEM173 activation/lipid peroxidation.^70^, ^71^, ^72^, ^73^ In addition, magnesium, cAMP and vitamin E can protect against sepsis by blocking GSDMD‐induced pyroptosis^70^, ^74^, ^75^ These results provide novel insight into the role of GSDMD‐triggered pyroptosis in sepsis, thereby revealing some potential targets for therapeutic intervention for lethal infection.

### Multiple sclerosis

Multiple sclerosis, a chronic inflammatory disease of the central nervous system (CNS), is characterised by inflammatory demyelination, chronic axonal damage and neurodegeneration. Previously, NLRP3 inflammasome, caspase‐1 and IL‐1β all have been implicated in the pathogenesis of MS.^76^, ^77^ In a recent study, Li *et al*.^78^ identified GSDMD is a driver of experimental autoimmune encephalomyelitis (EAE) in mice, an animal model of MS. Mechanistically, GSDMD‐mediated pyroptosis in peripheral myeloid cells promotes the priming, differentiation and activation of T cells in the secondary lymphoid organs and then recruits T‐cell infiltration into the CNS, ultimately inducing neuroinflammation in EAE. Collectively, this finding represents the first indication of a possibly essential role of GSDMD in the pathogenesis of MS. Consequently, targeting GSDMD‐mediated pyroptosis might be a potential therapeutic strategy for MS treatment.

### Systemic sclerosis

Systemic sclerosis is an autoimmune rheumatic disease characterised by excessive production and accumulation of fibrosis in the skin and internal organs and by injuries to small arteries. Although genetic underlying of SSc is suspected, key susceptibility gene of SSc remains unknown. Using transethnic meta‐analysis of genome‐wide association studies (GWASs) in the Japanese and European populations and two replication studies, Terao *et al*.^79^ identified rs3894194, a missense mutation of GSDMA, as a novel susceptibility locus for SSc. Interestingly, they found the signal of rs3894194 is enriched in keratinocytes and fibroblasts. These findings may suggest the potential pathogenic role of keratinocytes and fibroblasts in SSc. Further functional annotation of the rs3894194 allele in SSc is needed in the future.

### Inflammatory bowel disease

Inflammatory bowel disease entails diseases characterised by chronic inflammation of the GI tract, including Crohn's disease and ulcerative colitis. Genetic factors, dysbiosis of the microbiome, dysregulation of the immune system and environment may all contribute to the pathogenesis of IBD. However, the exact cause of IBD is largely unknown. Many studies aim to identify genetic risk factors for IBD. Intriguingly, GSDMA and GSDMB are identified to be IBD susceptibility genes.^80^, ^81^, ^82^ However, the mechanism by which GSDMA and GSDMB affect IBD onset and/or progression still requires further experimental investigation.

Recently, it was shown that GSDMD‐dependent pyroptosis in intestinal epithelial cells, which is triggered downstream of never in mitosis gene A (NIMA)‐related kinase 7 (NEK7)‐mediated NLRP3 inflammasome activation, is implicated in the pathogenesis of IBD *in vitro* and *in vivo*.^83^ This study provides the first experimental evidence that may help in understanding how GSDMD‐mediated pyroptosis may contribute to IBD.

### Rheumatoid arthritis

Rheumatoid arthritis, a disabling autoimmune disorder, is characterised by chronic inflammation, stiffness and destruction of the synovial joints. While the cause of RA is not clear, again genetic factors are believed to be involved in the aetiology of RA. To unravel the complex genetic architecture of RA, a multiethnic GWAS and bioinformatic analysis were used to identify the most likely causal variants and genes in RA. These researches identified that GSDMB locus is associated with risk of RA.^84^ Further studies showed that CD4^+^ and B lymphocytes, the important inflammatory executors in RA, exhibit overlapping expression quantitative trait locus at GSDMB locus.^85^ These data extend our understanding of the landscape of GSDMB in early RA, especially in several critical lymphocyte populations of early RA.

A recent study from Zhang and his team demonstrated that complement C1q synergises with pentraxin 3 (PTX3) in promoting NLRP3 overactivation, ultimately resulting in GSDMD‐mediated monocyte pyroptosis and excessive inflammatory cytokine release in RA. Additionally, the release of inflammatory cytokines, such as IL‐6, in turn, drives PTX3 plus C1q‐induced GSDMD‐dependent monocyte pyroptosis in a positive feedback.^86^ These findings further explore the pathogenic effects of gasdermin family on RA, suggesting a potential novel therapeutic strategy by targeting pyroptosis in RA.

### Familial Mediterranean fever

Familial Mediterranean fever, the most common monogenic autoinflammatory disease worldwide, is characterised by recurrent fever, abdominal pain, headache, rash, serositis, arthritis and dermal manifestations. It is caused by missense mutations in *MEFV* gene, which results in exaggeratedly activating the pyrin inflammasome.^87^, ^88^ However, the pathophysiologic mechanisms driven by excessive activation of pyrin inflammasome in FMF are incompletely understood. Recently, Kanneganti *et al*. demonstrated that GSDMD‐dependent pyroptosis, which is activated downstream of pyrin inflammasome, contributes to the autoinflammation‐associated growth retardation, anaemia, neutrophilia, cytokine production and tissue damage in *Mefv* ^V726A/V726A^ FMF mouse model. Importantly, deletion of GSDMD *in vivo* fully rescued these autoinflammation‐associated features.^89^ These findings identify GSDMD‐mediated pyroptosis is critical for autoinflammatory pathology in FMF, providing a potential therapeutic target for the treatment of FMF.

### Type 1 diabetes

Type 1 diabetes, which is characterised by insulin deficiency, is a common autoimmune disorder that arises from the action of multiple genetic and environmental risk factors. Chromosome 17q21, containing a cluster of five genes [GSDMA, GSDMB, ORM1‐like 3 (ORMDL3), IKAROS family zinc finger 3 (IKZF3) and zona pellucida‐binding protein 2 (ZPBP2)], was first linked to susceptibility to T1D in 2009.^90^, ^91^, ^92^ Then, Ayabe* et al*.^93^ further clarified the variant rs2290400, which is a non‐coding SNP located in intron 3 of GSDMB, functions as a T1D susceptibility allele in Japanese children. Importantly, this risk allele of rs2290400 in GSDMB probably determines the risk of T1D predominantly in early childhood. These studies may potentially shed light on the pathogenesis of T1D, especially the early childhood T1D. Further function study is needed to explore how the variant rs2290400 in GSDMB contributes to the early‐onset T1D in children.

## Therapeutic targeting of GSDMD

As gasdermin‐induced pore formation plays a pivotal role in sepsis and numerous autoinflammatory diseases, there is increasing interest to develop small molecule inhibitors targeting GSDMD and other gasdermin family members.

Since Cys191/192 (human/mouse) of GSDMD is the critical residue for oligomerisation during pyroptosis‐related pore formation,^45^ targeting Cys191/192 might be an attractive pharmacological strategy to block GSDMD‐induced pyroptosis. Rathkey and his group identified necrosulphonamide (NSA) to bind directly to GSDMD via Cys191 and therefore to inhibit GSDMD‐mediated pyroptosis downstream of inflammasome activation. In addition, NSA treatment significantly increased survival in LPS‐induced sepsis *in vivo*, suggesting that GSDMD inhibitors may show clinically efficacy in treating sepsis.^94^

In parallel, Hu *et al*.^95^ discovered disulphiram (a drug used to treat alcohol addiction) and Bay 11‐7082 (a previously identified NF‐κB inhibitor) to potently inhibit GSDMD pore formation in liposomes and inflammasome‐mediated pyroptosis via covalently modifying Cys191/192 (human/mouse) of GSDMD. In line with Hu's^95^ study, mice administered with disulphiram are protected from LPS‐induced septic shock and MOG_35–55_ peptide‐triggered EAE^78^ *in vivo*. These results also demonstrated that GSDMD might be an alternative target for the treatment of sepsis, MS and potentially additional inflammatory diseases.

In addition, using a chemical screen of 182 710 small molecules and proteomics, a small molecule LDC7559 was identified to specifically block PMA‐induced GSDMD‐dependent NET formation.^16^ Mechanistically, LDC7559 strongly suppressed the release of myeloperoxidase and NE from granules, ultimately inhibiting NE‐cleaved GSDMD to execute NETosis upon PMA stimulation. This compound, or its derivatives, might be attractive lead compounds for targeting GSDMD activity.

## Conclusions and future perspectives

Understanding of pyroptotic cell death has rapidly progressed since the identification of GSDMD as a key mediator of pyroptosis. Initially, pyroptosis has been defined as a form of lytic cell death downstream of inflammatory caspase activation. Recent studies revealed that the active pro‐apoptotic caspases or granzymes are also involved in the progression of pyroptosis.^12^, ^13^, ^14^ Moreover, besides pyroptosis, gasdermins also participate in NETosis, secondary necrotic death and apoptosis.^7^, ^58^, ^61^ Further studies are needed to explore in detail the mode of activation of gasdermins and their precise role(s) in these diverse modes of cell death.

Accumulating evidence demonstrates that gasdermin‐mediated pyroptosis contributes to the pathology of several autoinflammatory disorders, including MS, SSc, IBD and RA.^96^, ^97^, ^98^, ^99^ However, the potential pathogenic role of pyroptosis in other autoimmune diseases, such as psoriasis, systemic lupus erythematosus and ankylosing spondylitis, remains unknown. It would be of great interest to further investigate whether gasdermin‐triggered pyroptosis contributes to these and other autoinflammatory diseases.

Recently, GSDMB, GSDMD and GSDME are described to be cleaved and activated by inflammatory/pro‐apoptotic caspases or granzymes. Nevertheless, we still know very little about GSDMA and GSDMC. GSDMA and GSDMC also contain the pyroptotic N‐terminal domain; however, the physiological or pathological signals that mediate GSDMA or GSDMC cleavage remain unclear. In addition, the role of GSDMA‐ and GSDMC‐mediated pore formation in biology and diseases is also still poorly understood.

The activation of GSDMD or GSDME downstream of active caspase‐3 has opposing consequences.^12^, ^14^, ^15^ It is therefore important to determine the structural effect(s) of active caspase‐3 on the distinct gasdermin proteins and the biological consequences hereof. Thus, future research should explore the balance of caspase‐3 in inhibiting and activating pyroptosis and apoptosis, respectively, and how the crosstalk between these pathways regulates the immune response to microbial infection *in vivo*.

As previously mentioned, the GSDMD^NT^ fragment can directly and efficiently kill intracellular and extracellular bacteria by binding to specific lipid classes present in bacterial membranes and therefore can form transmembrane pores in these bacterial membranes.^45^, ^50^ Given the efficient killing bacteria properties, the pyroptotic GSDMD^NT^ fragment may be considered a novel antimicrobial agent to treat *E. coli*, *Mycobacterium* and δ‐proteobacterium *Bdellovibrio bacteriovorans* infections. In addition, this gasdermin‐derived pyroptotic fragment might be considered relatively safe since its ability to form pores is restricted by the lipid‐binding properties and thus the inner leaflet of mammalian cytomembranes. However, distinct bacteria compose a large diversity of structural lipids in their bacterial membrane.^69^ For example, the membrane of *Campylobacter jejuni* is enriched with phosphatidylethanolamine (PE) and phosphatidylglycerol (PG).^100^ The major membrane lipids of *Borrelia burgdorferi* are PG, phosphatidylcholine (PC) and glycolipids (GLs).^101^, ^102^ Additionally, *Porphyromonas gingivalis* accumulates PE, PG and sphingolipids in the membrane.^103^ Furthermore, the membrane of *Clostridium perfringens* is formed by PG, PE, alanyl‐PG (APG) and lysyl‐PG.^104^, ^105^ As a result of the diversity of bacterial membrane lipids and the selective lipid‐binding preferences of GSDMD^NT^ fragment, it will be not come as a surprise that pathogens such as *Campylobacter jejuni*, *B. burgdorferi*, *P. gingivalis* and *C. perfringens* are resistant to GSDMD killing.

Finally, it should be noted that in order to become effective, the GSDMD^NT^ fragment should be able to reach the membrane compartment where it can bind and form pores. As such, the bacterial wall may impose a physical barrier that will not be passed passively, and thus, the effective use of GSDMD^NT^ fragment should be accompanied by treatments that enable the crossing of the GSDMD^NT^ fragment over the bacterial wall.

Growing evidence indicates that distinct gasdermin proteins display a cell‐type and tissue‐specific expression pattern. Although mechanism of gasdermin activation and participation in the different modes of cell death display common features for all gasdermins, there are also clear differences as described here. Therefore, it will be very interesting to determine the cell‐type and tissue‐specific role(s) of the different gasdermins as this may help to explain their varying role in the different diseases. Future studies should also further characterise the function of different gasdermins in immune cells and non‐immune cells during infections and diseases. For example, GSDMB, which is highly expressed in oesophageal and gastric tissues, may be associated with IBD susceptibility. However, the detailed cellular mechanism by which GSDMB contributes to IBD progression remains largely unknown. Further studies are required to investigate whether GSDMB‐mediated pyroptosis contributes to IBD.

The field of microbe–host interplay has gained much attention in recent years. Increasing evidence suggests that microbial dysbiosis in skin or gut is oftentimes involved in diverse inflammatory diseases, including psoriasis, atopic dermatitis and IBD.^106^, ^107^, ^108^, ^109^ Given the high expression and the host‐defensive role of GSDMA, GSDMC and GSDME in the skin, it is therefore reasonable to speculate that GSDMA, GSDMC and GSDME may exert critical physiological functions in skin. Additionally, GSDMA, GSDMC and GSDME may act as a bridge between microbiome and skin inflammatory diseases.

Since GSDMD drives a variety of inflammatory diseases, the identification of drugs targeting GSDMD and other gasdermin family members is attractive. The newly found GSDMD inhibitors do open novel therapeutic avenues for sepsis, MS and other autoinflammatory diseases. However, NSA, disulphiram and Bay 11‐7082 likely lack sufficient specificity and will also inhibit other proteins in the pyroptotic pathway,^94^, ^95^ or display off‐target effects. Further research will be needed to identify novel GSDMD inhibitors with high specificity. The recent descriptions of the crystal structure of the various gasdermin proteins may offer a structural basis to design specific inhibitors of GSDMD in future.^3^, ^4^, ^110^

## Author Contributions

**Lipeng Tang:** Conceptualization; Writing‐original draft; Writing‐review & editing. **Chuanjian Lu:** Writing‐review & editing. **Guangjuan Zheng:** Writing‐review & editing. **Boudewijn MT Burgering:** Writing‐review & editing.

## Conflict of Interest

The authors declare no conflict of interest.
